# Intranasal dexmedetomidine and intravenous ketamine for procedural sedation in a child with alpha-mannosidosis: a magic bullet?

**DOI:** 10.1186/s13052-019-0711-1

**Published:** 2019-09-03

**Authors:** Matteo Trevisan, Sara Romano, Egidio Barbi, Irene Bruno, Flora Maria Murru, Giorgio Cozzi

**Affiliations:** 1 Department of Medicine, Surgery, and Health Sciences, University of Trieste, Trieste, Italy; 2 Department of Radiology, Institute for Maternal and Child Health - IRCCS “Burlo Garofolo”, Trieste, Italy; 3 Department of Pediatrics, Institute for Maternal and Child Health - IRCCS “Burlo Garofolo”, Trieste, Italy. Via dell’Istria 65/1, 34131 Trieste, Italy; 4 Pediatric Emergency Department, Institute for Maternal and Child Health - IRCCS “Burlo Garofolo” – Trieste, Italy. Via dell’Istria 65/1, 34131 Trieste, Italy

**Keywords:** Dexmedetomidine, Ketamine, Procedural sedation, Alpha-mannosidosis

## Abstract

**Background:**

Procedural sedation is increasingly needed in pediatrics. Although different drugs or drugs association are available, which is the safest and most efficient has yet to be defined, especially in syndromic children with increased sedation-related risk factors.

**Case report:**

we report the case of a five-year-old child affected by alpha-mannosidosis who required procedural sedation for an MRI scan and a lumbar puncture. We administered intranasal dexmedetomidine (4 μg/kg) 45 min before intravenous cannulation, followed by one bolus of ketamine (1 mg/kg) for each procedure. The patient maintained spontaneous breathing and no desaturation or any complication occurred.

**Conclusion:**

intranasal dexmedetomidine and intravenous ketamine could be a feasible option for MRI and lumbar puncture in children with alpha-mannosidosis needing sedation.

## Introduction

Procedural sedation is an emerging cornerstone in pediatrics aiming to control pain, decrease fear and emotional response when immobility is required or during painful procedures. The ideal sedative drug should have a prompt onset of action, be easy to administer, with a short elimination half time, offering efficacious pain relief without side effects. Particular attention is required when procedural sedation is needed in patients with disabilities associated with specific genetic disease or neurologic impairment. These patients present reduced communicative and expressive skills which reduce pain recognition and cooperation and they experience more pain and distress when compared to healthy peers [1]. Therefore, they are frequently candidates for sedation, which is a challenge due to their comorbidities, which cause increased sedation-related risk. For example, children affected by storage diseases usually present higher risks due to hypotonic pharynx and soft tissue thickness, neck stiffness, atlantooccipital instability and cardiopathies. They are predisposed to serious anesthetic complications, such as airway collapse with challenging airway management and respiratory depression with difficult ventilation and oxygenation. In addition, their atlanto-occipital instability can lead to spinal compression and subsequent paralysis. In these fragile patients, anesthetic drugs such as propofol and midazolam can facilitate cardio-respiratory complications even within standard doses, affecting respiratory drive and airway shape. For this reason, the association of dexmedetomidine and ketamine, maintaining active upper respiratory reflexes and minimally impacting on airway shape, could allow safer sedation.

Finally, venous access may be troublesome, scaring and painful for these children, who often require repeated hospital admissions and procedures, so that a sedation sequence based on an intranasal premedication, facilitating cannulation without putting the patient at risk, may offer further advantages.

## Case report

We report the case of a five-year-old child, affected by alpha-mannosidosis requiring a brain magnetic resonance (MRI) and a lumbar puncture during a planned follow-up for his enzymatic substitutive treatment. He had received a genetic diagnosis, showing mutation of the gene MAN2B1, because of coarse facial features and mild mental retardation. In addition, he developed psychomotor and speech retardation, recurrent respiratory infections, inguinal hernia, vertebral body listhesis and bilateral neurosensorial deafness. No hepatic, splenic, cardiac or bone marrow involvement were detected. Because of the difficult airway management and a history of snoring sleep, notwithstanding a previous adeno-tonsillectomy procedure, we decided to associate ketamine and dexmedetomidine for the procedural sedation. Clear fluids were offered up to 2 hours before the procedure. 90 min before the scheduled cannulation, the child received a topical anesthetic cream on two possible sites and 45 min before the cannulation attempt, intranasal dexmedetomidine (4 μg/kg by mean of a Mucosal Atomizing Device) was administered. The emla cream was removed 20 min before the cannulation attempt, in order to avoid stimulating the child by stripping the dressing from the skin just before venipuncture, targeting a good sedation level before the puncture.

This premedication allowed to perform a painless and fearless intravenous access. A bolus of intravenous ketamine (1 mg/Kg) was administered twice, before both procedures, to diminish the estimated risk of about 30% of sedation failure with dexmedetomidine alone for MRI [2] and the expected lumbar puncture’s pain.

The patient was monitored with end-tidal carbon dioxide (EtCO_2_) nasal cannula and pulse oximetry. During the MRI scan the patient lied down with the head slightly rotated to achieve the best airways’ patency, maintaining spontaneous breathing without desaturations or complications (SpO_2_ 95–98%, HR 93–97 and EtCO_2_ 40–45).

**Fig. 1:**
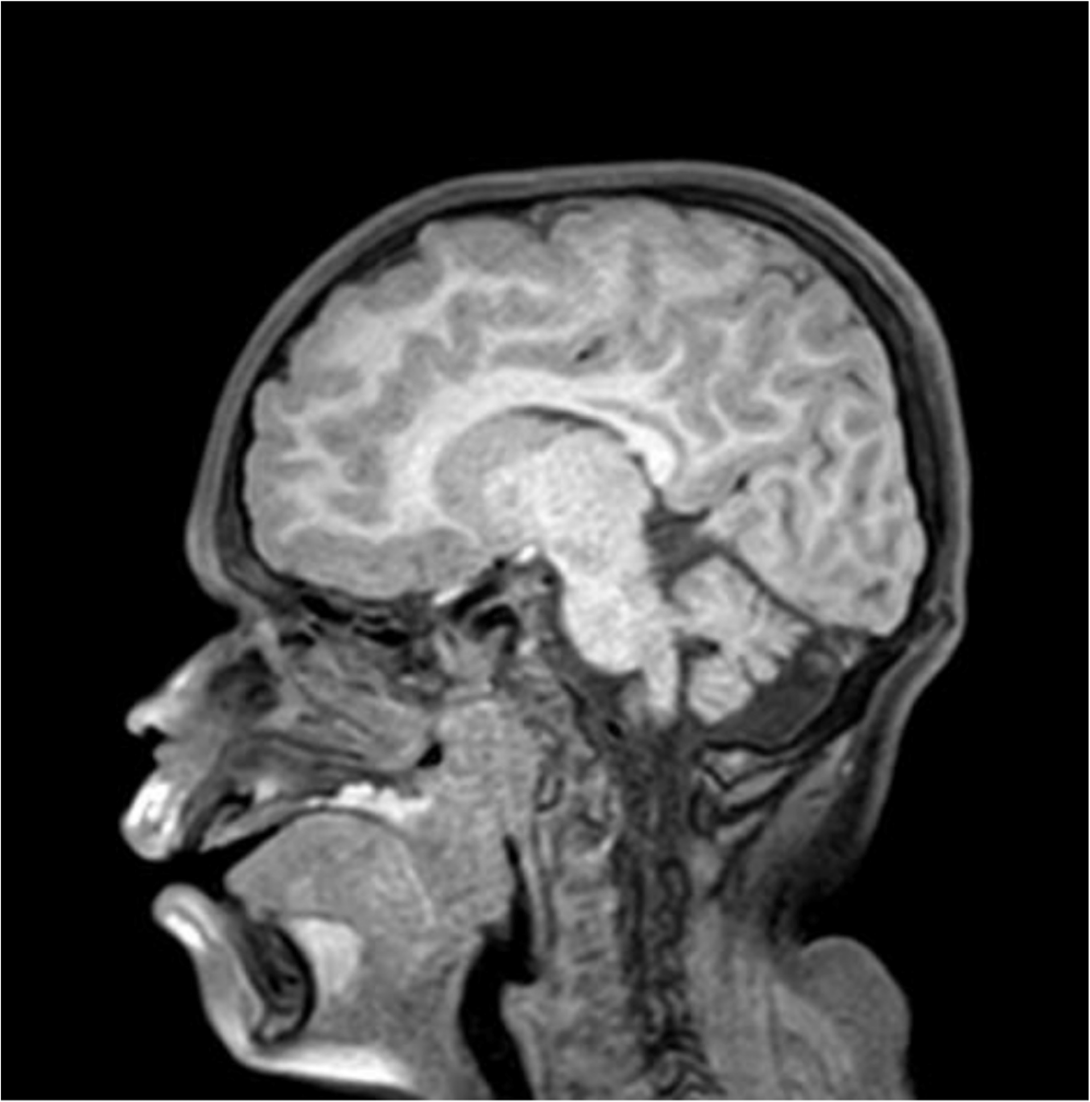
MRI scan showing a very narrow air column in the larynx and pharynx space along with the significant adenotonsillay hypertrophy

Findings of the MRI evaluation showed a very narrow air column in the larynx and pharynx space, confirming the high risk of an upper respiratory collapse (Fig. 1.) The child did not require supplemental oxygen or any intervention. Further, he underwent lumbar puncture after administration of another bolus of ketamine at the same dose, lying on a side, without any complication. Both procedures lasted a total of 55 min and an hour after, the patient awoke in a well-appearing state.

## Discussion

While sedation in high-risk patients should be managed only by experienced pediatric anesthesiologists or intensivists, the association of dexmedetomidine and ketamine has already been reported as a highly efficacious and safe option also in the setting of procedural sedation for children without risk factors, in the general perspective of adequately trained and skilled pediatric sedation providers.

Alpha-mannosidosis is a rare autosomal recessive lysosomal storage disorder with an estimated prevalence of one in 500,000–1,000,000 live births [3]. A decreased alpha-mannosidase activity results in impaired glycoprotein degradation in the lysosomes, as well as compromised cellular function and apoptosis [4]. The high prevalence of airway obstruction and restrictive pulmonary disease, cardiac impairment and cervical spine issues put these patients to a high anesthetic risk. The upper airways can be narrowed due to the accumulation of mannose-rich oligosaccharides causing macroglossia, adenotonsillary hypertrophy and thickened soft tissues in the laryngopharynx. Other typical features are deformities of the skull or spine, flattened nasal bridge, short neck, high anterior larynx, mandibular abnormalities or abnormal cervical vertebrae [5]. Aiming to limit any possible effects on respiratory functions, dexmedetomidine and ketamine constitute a good combo.

Ketamine is a dissociative and sedative drug, frequently used for painful procedures, because of its potent analgesic and amnestic effect with minimal respiratory or circulatory depression [6]. It can be administered either via intravenous, intramuscular, intranasal or oral routes. Nevertheless, the oral and intranasal ways display a low bioavailability and, in order to achieve adequate sedation, much higher doses are required [7]. Most common side effects are mild hypertension and tachycardia, emesis (8–25%), hypersalivation, hallucination, psychotomimetic effect (7%) and laryngospasm (0.3%) [8]. Such adverse effects can be managed with administration of ondansetron for vomiting and positive airway pressure to overcome laryngospasm. Hypersalivation usually is self-limited, not requiring any medication [9]. Ketamine, when used by pediatricians, has an impressive safety record in the literature and should be considered as a standard of care in the pediatric emergency department [10].

Dexmedetomidine is α_2_-adrenoreceptor agonist that induces sedation, anxiolysis and mild analgesia, without respiratory depression [11]. Its main side effects are bradycardia and hypotension, usually not requiring any medication. While the intravenous administration is more used in pediatric intensive care, it has been demonstrated that the intranasal route has a good bioavailability, decreasing children’s pain and distress and inducing sedation as an effective premedication [12, 13]. Prophylactic use of atropine should be avoided when using dexmedetomidine, due to the high risk of blood pressure hypertension [14].

The most frequently used premedication sedative in children is midazolam. Midazolam has untoward side effects such as paradoxical reactions, respiratory depression in patients with specific risk factors, amnesia and unpleasant taste. Moreover, when it is administered via intranasal route, many patients complain of nasal burning and gagging even after lidocaine premedication. Dexmedetomidine is an odorless, colorless and tasteless solution, its intranasal use is painless and could be an innovative approach for pediatric premedication, in particular for a successful venous cannulation [15]. As a matter of fact, a meta-analysis of thirteen randomized controlled trials about premedication with dexmedetomidine in pediatric patients revealed the superiority of the latter on midazolam in promoting preoperative sedation and decreasing postoperative pain [16]. Intranasal dexmedetomidine, when used as the sole sedative drug for pediatric MRI, requires a rescue treatment in 30% of cases. When intranasal dexmedetomidine is associated with oral midazolam (0.5 mg/kg) the success rate significantly increases, but still at the age of 5 years, a 10% of patients will require an additional treatment to adequately perform MRI imaging [2].

As for ketamine, substantial data about safety of non-anesthesiologist managed dexmedetomidine are available [17, 18].

Several authors proposed dexmedetomidine and ketamine as a successful option to obtain a deeper and painless sedation (Tab. 1). Their main advantage consists in a deep and painless sleep, maintaining the respiratory drive and active upper airway reflexes. Indeed, many case reports described a successful and safe use of dexmedetomidine and ketamine for procedural sedation, also when analgesia and autonomous ventilation are required [26, 28]. For this reason, their administration could be particularly safe when the maintenance of spontaneous ventilation is a priority, such as patients with cardiorespiratory comorbidities or high risk airway obstruction [19]. In addition, their pharmacological association may prevent each other’s adverse events, having limited effects on respiratory function. Dexmedetomidine counteracts tachycardia, hypertension and emergence agitation from ketamine, while the latter prevents bradycardia and hypotension which has been reported with dexmedetomidine [29]. The most frequent drawbacks of the association are nausea and vomiting, preventable with ondansetron’s administration, and a long recovery time.

**Table 1:** Association of dexemedetomidine and ketamine in literature

Study (year)	Study design	Population	N°	Drugs and administration route	Results/side effects
Luscri N. et Tobias J.D. (2006) [19]	Case report	Children with trisomy 21 and OSA undergoing MRI	3	Ketamine (iv) 1 mg/Kg and dexmedetomidine (iv) 1 μg/Kg and maintained by a continuous infusion of dexmedetomidine (1 μg/Kg/h)	Effective sedation without hemodynamic or respiratory effects
Kandil,A.et al. (2016) [20]	Retrospective	Children with refractory OSA undergoing DISE	59	Group DK: Dexmedetomidine (iv) 1.9 μg/Kg (1.6 μg/Kg/h) + Ketamine (iv) 2.0 mg/Kg; Group P: Propofol (iv) 1.8 mg/Kg (248 μg/Kg/min); Group SP: Propofol (iv) 1.8 mg/Kg (192 μg/Kg/min) + Sevoflurane	Patients in Group DK had significantly fewer desaturations to< 85% during DISE compared to Group P. Patients in Group DK had significantly more successful completion of DISE (100% Group DK, 92% Group P, and 79% Group SP) as compared to Group SP
Kako H. et al. (2014) [21]	Prospective	Patients with Duchene muscular dystrophy undergoing muscle biopsy	19	Group A: Dexmedetomidine (iv) (1 μg/kg over 3 min, 1 μg/kg/h) + ketamine (iv) (1 mg/kg) Group B: Dexmedetomidine (iv) (0.5 mcg/kg over 3 min, 0.5 mcg/kg/h) + ketamine (iv) (1 mg/kg)	Both groups had effective sedation; a decrease in heart rate occurred after the loading dose of dexmedetomidine in both groups; shorter recovery time in group B
Goyal R. et al. (2012) [22]	Case series	Children 2–12 years old undergoing upper gastrointestinal endoscopy	46	Dexmedetomidine 1 μg/kg (iv) + ketamine (iv) 2 mg/kg	Adequate sedation without cardio-respiratory depression. Hiccup, vomiting and increased salivation were the most frequent side effects
Joshi VS. et al. (2017) [23]	RCT	Children undergoing cardiac catheterization	60	Premedication with glycopyrrolate and midazolam iv (0.05 mg/kg). Dexmedetomidine iv 1 μg/kg over 10 min (0.5 μg/kg/h) + ketamine iv 1 mg/Kg (1 mg/kg/h) vs propofol iv 1 mg/kg (100 μg/kg/hr) + ketamine iv 1 mg/kg/hr. (1 mg/kg/hr).	Dexmedetomidine and ketamine are safe and effective sedative drugs, without major side effects but long recovery time
Qiao H. et al. (2017) [24]	RCT	Children 2–5 years old undergoing premedication in eye surgery	135	2.5 μg/kg in dexmedetomidine vs 3 mg/kg os ketamine and 2 μg/kg in dexmedetomidine vs 6 mg/kg os ketamine 30 min before surgery.	Combination of in dexmedetomidine and os ketamine produces satisfactory separation from parents and more successful venous cannulation. Postoperative vomiting, (*p* = 0.0041) respiratory-related complications during the perioperative period (*p* = 0.0032) and postoperative psychological/psychiatric adverse events (*p* = 0.0152) were detected in the ketamine group.
McVey J. and Tobias D. (2010) [25]	Retrospective	Children 2–9 years old undergoing lumbar puncture for spinal anesthesia	12	Ketamine iv (2 mg/kg) and dexmedetomidine iv (1 μg/kg) over 3 min + dexmedetomidine (2 μg/kg/hr. for the first 30 min and 1 μg/kg/hr. until the end).	Effective sedation with limited effects on cardiovascular and ventilator function
Yang F. et al. (2019) [26]	Retrospective	Children undergoing procedural sedation	17,948	Ketamine 1 mg/Kg (in) + dexmedetomidine 2 μg/kg (in)	The rate of in sedation success was 93%, in sedation rescue was 1.8% and in sedation failure was 5.2%. Incidence of adverse events was low (0.58%). Postoperative nausea and vomiting were the most common (0.3%)
Jia JE et al. (2013) [27]	RCT	Children 2–6 years old	160	Group 1: 1 μg/kg in dexmedetomidine with 3 mg/kg os ketamine; Group 2: 1 μg/kg in dexmedetomidine with 5 mg/kg os ketamine; Group 3: 2 μg/kg in dexmedetomidine with 3 mg/kg os ketamine; Group 4: 2 μg/kg in dexmedetomidine with 5 mg/kg os ketamine.	Patients in Group 4 were significantly more sedated than those in Group 1 after 30 min (*p* = 0.036). A significantly higher proportion of patients in Group 3 (84%) and Group 4 (87%) accepted intravenous cannulation compared with those in Group 1 (40%) and Group 2 (54%) (*p* = 0.001).

MRI = magnetic resonance imaging; OSA = obstructive sleep apnea; DISE = drug-induced sleep endoscopy; in = intranasal; os = oral; iv = intravenous

Premedication with intranasal dexmedetomidine followed by intravenous ketamine may offer significant advantages, facilitating venous access in patients requiring painful procedures, in which the use of dexmedetomidine only could not guarantee an adequate level of pain control and sedation.

## Conclusion

This is the first case described in literature in which a child with alpha-mannosidosis successfully underwent procedural sedation with intranasal dexmedetomidine and intravenous ketamine.

This case highlights the advantages of the association of intranasal dexmedetomidine followed by intravenous ketamine. Further prospective studies should follow this proof of concept.

## Data Availability

No supporting data are available.

## Abbreviations

DISE: Drug-induced sleep endoscopy
DK: Dexmedetomine and ketamine
EtCO_2_: End-tidal carbon dioxide
HR: Heart rate
In: Intranasal
Iv: Intravenous
MRI: Magnetic resonance imaging
Os: Oral
OSA: Obstructive sleep apnea
P: Propofol
SP: Propofol and sevoflurano
SpO_2_: Oxygen saturation
